# fMR-adaptation indicates selectivity to audiovisual content congruency in distributed clusters in human superior temporal cortex

**DOI:** 10.1186/1471-2202-11-11

**Published:** 2010-02-02

**Authors:** Nienke M van Atteveldt, Vera C Blau, Leo Blomert, Rainer Goebel

**Affiliations:** 1 Maastricht University, Faculty of Psychology & Neuroscience, Dept of Cognitive Neuroscience, PO Box 616, 6200 MD Maastricht, the Netherlands; 2 Dept of Psychiatry, Columbia University College of Physicians and Surgeons & New York State Psychiatric Institute, 1051 Riverside Drive, NY 10032, New York, USA; 3 Netherlands Institute for Neuroscience, Meibergdreef 47, 1105BA Amsterdam, the Netherlands

## Abstract

**Background:**

Efficient multisensory integration is of vital importance for adequate interaction with the environment. In addition to basic binding cues like temporal and spatial coherence, meaningful multisensory information is also bound together by content-based associations. Many functional Magnetic Resonance Imaging (fMRI) studies propose the (posterior) superior temporal cortex (STC) as the key structure for integrating meaningful multisensory information. However, a still unanswered question is how superior temporal cortex encodes content-based associations, especially in light of inconsistent results from studies comparing brain activation to semantically matching (congruent) versus nonmatching (incongruent) multisensory inputs. Here, we used fMR-adaptation (fMR-A) in order to circumvent potential problems with standard fMRI approaches, including spatial averaging and amplitude saturation confounds. We presented repetitions of audiovisual stimuli (letter-speech sound pairs) and manipulated the associative relation between the auditory and visual inputs (congruent/incongruent pairs). We predicted that if multisensory neuronal populations exist in STC and encode audiovisual content relatedness, adaptation should be affected by the manipulated audiovisual relation.

**Results:**

The results revealed an occipital-temporal network that adapted independently of the audiovisual relation. Interestingly, several smaller clusters distributed over superior temporal cortex within that network, adapted stronger to congruent than to incongruent audiovisual repetitions, indicating sensitivity to content congruency.

**Conclusions:**

These results suggest that the revealed clusters contain multisensory neuronal populations that encode content relatedness by selectively responding to congruent audiovisual inputs, since unisensory neuronal populations are assumed to be insensitive to the audiovisual relation. These findings extend our previously revealed mechanism for the integration of letters and speech sounds and demonstrate that fMR-A is sensitive to multisensory congruency effects that may not be revealed in BOLD amplitude per se.

## Background

To adequately perceive and respond to the environment, our brain has to integrate information relayed by the different senses. For the integration of meaningful information, content-based associations are important to determine which inputs belong together [1,2], in addition to more basic binding cues like temporal and spatial coherence [3]. Content-based associations refer to inputs of different sensory modalities that closely correspond in content [1], in other words, are semantically matching or congruent [2].

Functional magnetic resonance imaging (fMRI) studies consistently propose the (posterior) superior temporal sulcus (STS) and gyrus (STG) as the key structure for integrating meaningful audiovisual information in humans ([4-7]; but see [8]). A still open question, however, is how content-based multisensory associations are encoded in STS/STG, as studies that compared brain activation to semantically matching (congruent) versus nonmatching (incongruent) inputs report inconsistent results. Although some fMRI studies report congruency effects in STS/STG [9,10], other studies do not [11-14], or to a much weaker extent than in "unisensory" auditory regions [15]. Some studies report effects in the opposite direction, i.e., stronger activation for incongruent than congruent multisensory information [8,16]. These discrepancies may in some cases be explained by different task demands. As we showed recently, the task to explicitly match audiovisual information may overrule perceptual congruency effects observed in passive viewing/listening conditions [14]. Also, the studies that report stronger activation for incongruent stimulus pairs presented the stimuli sequentially rather than simultaneously, indicating potential repetition suppression effects for congruent pairs (see below).

Importantly, the inconsistent findings on congruency effects may, at least partially, also be due to limitations inherent to the method of fMRI. Since the Blood Oxygenation Level Dependent (BOLD) signal reflects an averaged response over all (hundred thousands of) neurons in a voxel and its amplitude is subject to hemodynamic saturation effects [17,18], fMRI may lack the spatial precision and dynamic range to reflect differential neural responses to congruent and incongruent audiovisual information. Convergence and interaction of visual and auditory input on single neurons in monkey STS has been demonstrated by electrophysiological recordings [19,20], and, more recently, these interactions have been shown to depend on the congruency between both inputs [21]. Moreover, recent high-resolution fMRI evidence suggests that the human STS is composed of a patchy distribution of unisensory and multisensory neuronal subpopulations, at a resolution below the typical fMRI voxel size (millimeter range, [22]). The neuronal basis of this spatial layout has recently been provided by Dahl and colleagues [23]. These findings predict that at standard fMRI-resolution, some voxels in STS consist of a mixture of unisensory and multisensory subpopulations, others only of unisensory subpopulations. Since only the multisensory subpopulations within STS would be sensitive to audiovisual relatedness, potential congruency effects on the neuronal level have a high chance to be averaged out at the voxel level at standard resolution. Furthermore, even if the differential multisensory response is strong enough when averaged over all neuronal responses in a voxel, the BOLD response might saturate, i.e., lack enough dynamic range to reflect the different neuronal responses to congruent and incongruent information in its amplitude. As unisensory neurons also drive the BOLD response in a mixed voxel without being sensitive to the cross-modal relation, the putative selective response of multisensory neurons might disappear in the ceiling level of the fMRI response.

Here, we explored an alternative approach to study human multisensory integration of meaningful information by employing a variation of the fMR-adaptation (fMR-A) paradigm. fMR-A refers to a reduced fMRI signal to stimulus repetitions, and is based on the phenomenon of reduced neural activity to repetitions (repetition suppression) [24]. It hypothesizes that by targeting specific neuronal subpopulations within voxels, their functional properties can be measured at subvoxel resolution since it circumvents spatial averaging. Adaptation effects have robustly been demonstrated by single unit recordings, EEG and fMRI in many cortical regions [25]. The typical fMR-A procedure is to compare adaptation conditions in which repetitions of identical stimuli or stimuli with one property varied are presented, to a no-adaptation condition, in which different stimuli are presented sequentially (no repetitions). In voxels containing neurons that are responsive to the repeated stimulus, repetition of identical stimuli leads to a reduced fMRI signal relative to the unadapted response. Critically for studying the functional properties of the adapted neuronal subpopulation, repeated stimuli with one property varied are presented, and the effect on adaptation strength is assessed. If adaptation remains (i.e., the fMRI signal stays low), the adapted neurons are assumed to be insensitive to the manipulated property. In contrast, an increased ("recovered") fMRI signal indicates sensitivity to the varied property as neurons no longer stay adapted and other neurons will be activated. Some studies applied a similar approach to study cross-modal processes, for instance the neural coding of audiovisual speech [26] and visual-tactile object processing [27]. Also, the studies of Noppeney et al. [16] and Hocking et al. [8] presented the cross-modal stimulus pairs sequentially; therefore, the weaker response to congruent pairs they report may be due to repetition suppression.

**Figure 1:**
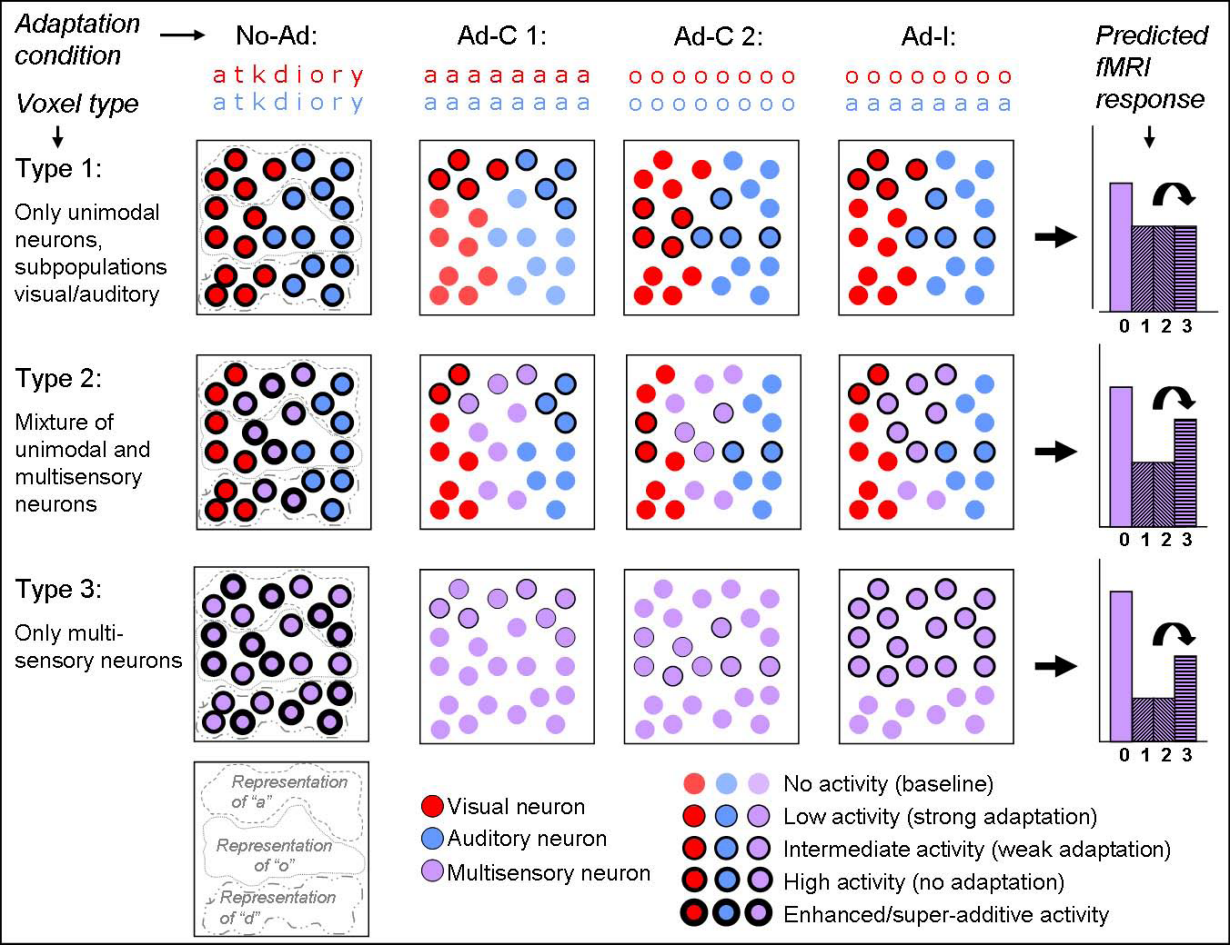
**Schematic of predictions tested in the present adaptation study - from neuronal to voxel-level responses**. The top row shows examples of the experimental conditions (red: visual stimuli, blue: auditory stimuli). Rows 2-4 show schematic illustrations of three possible voxel types in multisensory STS: containing only unisensory subpopulations (type 1), a mixture of uni- and multisensory populations (type 2) or only multisensory neurons (type 3). All circles represent one neuron (visual, auditory or audiovisual), the thickness of the lines indicate the neuron's activity (see index bottom right). Activation/adaptation strength is predicted for all neurons in the three voxel types for the different experimental conditions, and the resulting predicted fMRI response of the voxel in it's entity is shown in the column on the right (X-axis: 0 = No-Ad; 1 = Ad-C 1; 2 = Ad-C 2; 3 = Ad-I). Importantly, the relative activity for the Ad-C vs. Ad-I (marked by the black arrow) will distinguish between voxels containing multisensory neurons (type 2 or 3 → differential adaptation) or voxels consisting only of unisensory neurons (type 1 → identical adaptation). Hypothetical representations of three different letters ("a", "o" and "d") are indicated by encircled regions. To ensure equal averaged adaptation strength for different letter exemplars (Ad-C 1 vs Ad-C 2), each letter and each speech sound exemplar was equally often presented in congruent and incongruent audiovisual combinations (see also figure 2).

**Figure 2:**
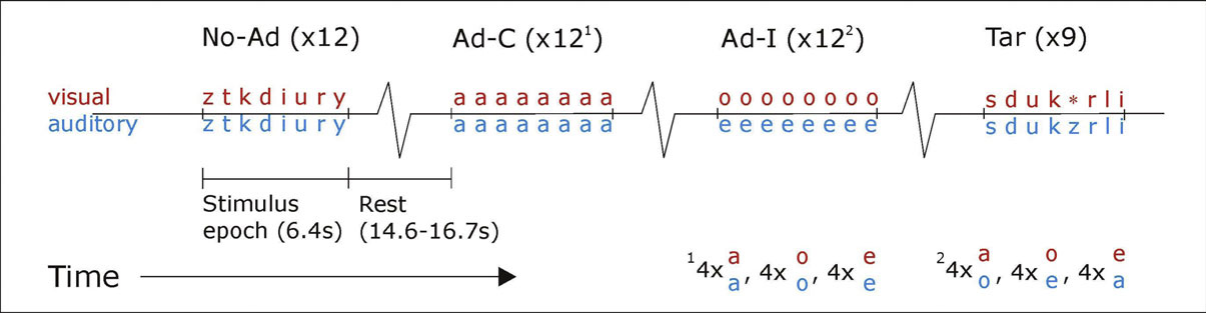
**Schematic of stimulus presentation and timing within one experimental run**. No-Ad: No-adaptation; Ad-C: Adaptation-congruent; Ad-I: Adaptation-incongruent; Tar: epoch including a target. Note that across adaptation conditions, each letter and each speech sound exemplar was equally often presented in congruent and incongruent audiovisual combinations. Subjects did not know when targets would appear; the purpose of the task was to obtain equal attention levels for the different conditions.

In the present study, we aimed to address the still open question of how multisensory content relatedness is encoded in the human superior temporal cortex (STS/STG) using an fMR-A design. As multisensory fMR-A designs using sequential presentation of different modalities as varied property are not straightforward (see [28] and "Discussion"), we used fMR-A in a slightly different way. We presented epochs of repeated identical audiovisual (AV) stimuli (letter-sound pairs) and varied the associative relation (congruency) between the auditory and visual inputs, but across blocks rather than within (see figures 1 and 2). There were two adaptation conditions: adaptation-congruent (Ad-C) epochs in which we presented corresponding letter-sound pairs, and adaptation-incongruent (Ad-I) epochs in which unrelated pairs were presented (while sequential AV-pairs were always identical). As the no-adaptation condition, we presented epochs of different AV-pairs.

We predicted that if multisensory neurons in STS/STG encode audiovisual content relatedness, adaptation in voxels containing multisensory neuronal subpopulations will be affected by the manipulated AV-relation, i.e., adapt stronger to repeated congruent than to repeated incongruent pairs. Furthermore, unisensory subpopulations are assumed to respond only to their preferred modality and therefore predicted to be insensitive to the AV-relation, and thus would adapt equally strong to repetitions of congruent and incongruent AV-pairs. This design therefore has the potential to distinguish between: 1) Voxels consisting only (or dominantly) of unisensory subpopulations, which will not show different adaptation (type 1 in figure 1); and 2) Voxels in which at least a strong subpopulation of multisensory neurons is present, which is sensitive to the associative relation between the auditory and visual stimuli, and will therefore show differential adaptation (type 2 & 3 in figure 1). Moreover, in comparison with conventional stimulus presentation, the present adaptation design is predicted to be more sensitive to reveal congruency effects because they will not occur within the ceiling range of the BOLD response.

## Methods

### Subjects

Sixteen healthy volunteers (6 male, mean age 22.8, range 19-32) participated in the present study. All subjects were recruited from an academic environment and had no history of reading/language problems or neurological/psychiatric disorders. All were right-handed Dutch native speakers, had normal or corrected-to-normal vision and normal hearing capacity. Subjects gave informed written consent and all procedures were approved by the local ethics committee (Faculty of Psychology, Maastricht University).

### Stimuli

Stimuli were pairs of letters (Visual, V) and speech sounds (Auditory, A) that were presented simultaneously (vowels a, e, i, y, o, u and consonants d, k, l, n, p, r, s, t, z). These stimuli showed multisensory integration effects in superior temporal cortex in our previous fMRI studies [9,13,15] and have the advantage that they can easily be presented in associated (i.e., congruent) and non-associated (incongruent) combinations. Speech sounds were presented phonetically (not letter names) and were digitally recorded (sampling rate 44.1 kHz, 16 bit quantization) from a female native Dutch speaker. Recordings were band-pass filtered (180 - 10000 Hz) and resampled at 22.05 kHz. Average duration of the speech sounds was 352 (±5) ms, average sound intensity level was approximately 70 dB SPL. White lower case letters (typeface "Arial") were presented for 350 ms on a black background. For the subject's task (see below), target stimuli were prepared consisting of a pure tone of 750 Hz (A, "beep") and a white star symbol of equal size as the letters (V, "star") and were also presented for 350 ms. Visual stimuli were projected onto a frosted screen positioned at the rear end of the scanner bore, and viewed by the participants through a mirror mounted onto the head coil. Auditory stimuli were presented with an MR-compatible Intercom Commander XG Audio System (Resonance Technologies Inc.). Stimuli were presented and synchronized with the scanner pulses using the software package "Presentation" (Neurobehavioral systems, USA). Participants' responses were registered by a handheld fiber-optic response system (LUMItouch fMRI Optical Response keypad, Photon Control, Burnaby, Canada).

### Adaptation procedure

Stimuli were presented in epochs of three main conditions: No-adaptation ("No-Ad"), Adaptation-congruent ("Ad-C") and Adaptation-incongruent ("Ad-I"). Each condition was repeated 12 times per run, two runs were acquired per subject. Subjects performed a target detection task (detect beeps and stars) to obtain equal attention levels during no-adaptation and adaptation epochs. In nine additional stimulation epochs (modelled as "Tar"), 3 of each main condition, one stimulus was randomly replaced by an auditory or visual target. Occurrence of targets was unpredictable for the subjects; their task was to press the button whenever they would hear a beep or see a star, so they had to attend all epochs. The epochs containing a target stimulus were included in the model but not further analyzed in the main statistical comparisons (see below).

In total, this resulted in 45 stimulation-epochs per run (36 without target, 9 with target), interspersed with rest/baseline periods in which only a white fixation cross was presented. In each stimulation epoch (6.4 s), 8 AV stimuli were presented sequentially at a rate of 1.25 Hz. The interval between the onset of 2 subsequent stimulation epochs was 21 or 23.1 s (10 or 11 scanning Repetitions Times (TR)). The rest periods between the stimulus epochs were 14.6 or 16.7 s, the first and last rest periods 18.9 s.

During No-Ad epochs, 8 *different *congruent AV stimuli were presented, randomly sampled from all consonant and vowel exemplars. During adaptation epochs, 8 *identical *AV stimuli were presented, pseudo-randomly sampled from the vowels, in congruent (Ad-C) or incongruent (Ad-I) combinations (see fig. 2). Because stimuli for Ad-C and Ad-I were sampled from a limited set (6 vowels), we selected three different vowels per run (e.g., a-e-o), and used the other three in the second run (e.g., u-i-y), to avoid unnecessary repetitions. The subset of vowels in each run was varied across subjects, as well as the AV-combinations presented in Ad-I. Importantly, in each run, each letter and each speech sound was equally often presented in a congruent (4×) as in an incongruent AV-pair (also 4×). For example, in a-e-o runs, we presented 4 a/a-epochs, 4 o/o, 4 e/e, 4 a/o, 4 o/e, and 4 e/a-epochs. This counterbalances potentially different unisensory response/adaptation strengths for the different stimulus exemplars across Ad-C and Ad-I. See figure 2 for a schematic of the stimulus presentation.

### fMRI scanning and analysis

**Figure 3:**
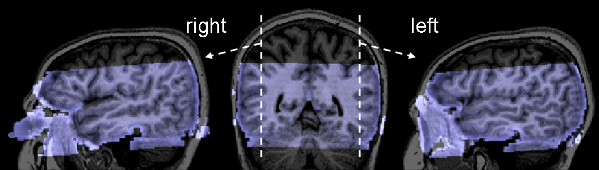
**Coverage of measured functional volumes**. The measured functional volume (transparent blue) overlaid on the anatomical image (gray) of a representative subject. The sagittal views (left and right brains) show that the temporal and occipital lobes are fully covered, including the entire STS on both sides. The coronal view (middle brain) shows the x-position of the sagittal views.

Imaging was performed on a 3 Tesla head scanner (Magnetom Allegra, Siemens Medical Systems, Erlangen, Germany) located at the Maastricht Brain Imaging Centre (M-BIC) in Maastricht, The Netherlands. A BOLD-sensitive EPI sequence was used for the functional scans (matrix 128 × 128, 30 slices, slice thickness 2.5 mm, FoV 256, voxel size 2 × 2 × 2.5 mm^3^, flip angle 90, TE 30 ms, slice-TR 70 ms, volume-TR 2100 ms). 480 volumes were acquired per run. To optimize spatial and temporal resolution, we scanned a slab of 7.5 cm positioned to cover the temporal and occipital lobes including the entire STS (instead of scanning whole-brain volumes). In figure 3, the coverage of functional volumes in a representative subject is shown. An intra-session high-resolution structural scan (voxel size: 1 × 1 × 1 mm^3^) was collected for each subject using a T1-weighted 3D ADNI MPRAGE sequence (TR = 2250 ms, TE = 3.6 ms, 192 sagittal slices).

BrainVoyager QX (Brain Innovation, Maastricht, The Netherlands [29]) was used for data analysis. Standard preprocessing was performed on the functional data: slice scan time correction, linear trend removal, temporal high pass filtering (≤3 cycles per time course), 3D motion correction (trilinear interpolation), and mean intensity adjustment (MIA). This latter step scales the global intensity of the repeatedly measured volumes to the average of the first volume; however, we only used the resulting time-courses of (global) volume intensity for data modelling (see below) to avoid wrongly correcting activation effects. Functional slices were co-registered to the anatomical volume using position parameters from the scanner and intensity-driven fine-tuning, and transformed into Talairach space. For data presentation, an averaged anatomical volume was created from the 16 individual anatomical volumes. All individual anatomical data-sets were segmented at the gray/white matter boundary using a semi-automatic procedure based on edge-preserving filtering and intensity histogram analysis, and the cortical surfaces were reconstructed. To improve the spatial correspondence between subjects' brains beyond Talairach space, the reconstructed cortices were aligned based on individual curvature information reflecting the gyral/sulcal folding pattern, using a "moving target" group averaging approach (cortex-based alignment, see [15,29]). Cortical functional time-series (sampled from -1 to 3 mm into gray matter; 0 = at vertex) were subsequently aligned across subjects using the resulting correspondence information. A shape-averaged (n = 16) folded cortical mesh was created for both hemispheres for projection of the cortex-based aligned statistical maps.

Functional time-series were analyzed using a random-effects multi-subjects general linear model (GLM). In the first level analysis, all experimental conditions in all subjects were modelled as separate predictors; in addition, the MIA time-course was added after z-normalization as a confound predictor to the design matrix of each run. The resulting GLM, thus, contained five predictors per subject: No-Ad, Ad-C, Ad-I, Tar and MIA. Predictor time-courses were adjusted for the hemodynamic response delay by convolution with a double-gamma hemodynamic response function. To explore adaptation effects, we calculated two second-level random-effects contrasts:

1) *general adaptation *[2*No-Ad vs. (Ad-C + Ad-I)]

2) *specific adaptation *[Ad-I vs. Ad-C]

The second contrast was critical to assess sensitivity to the AV-relation. Since our aim was to find voxels showing a different *adaptation *effect for Ad-I and Ad-C, and not a different response per se, we used the first contrast ("general adaptation") as a search constraint for the specific adaptation contrast: either by using a functional mask created from the general adaptation contrast (volume data), or as a conjunction of the first and second contrast (surface data). Volume data were modestly spatially smoothed using a Gaussian filter of 5 mm FWHM. Statistical maps shown in the volume domain were corrected for multiple comparisons using cluster-size thresholding [29,30]. Maps thresholded at an initial voxel-level p-value were submitted to a whole-data correction criterion based on the estimate of the map's spatial smoothness and on an iterative procedure (Monte Carlo simulation) for estimating cluster-level false-positive rates. After 1,000 iterations, the minimum cluster-size corresponding to a corrected false positive probability of 0.05 or less is applied to the statistical maps. Statistical maps on the surface are shown at the same t-values. In addition to statistical maps, averaged BOLD response time-courses for No-Ad, Ad-C and Ad-I were extracted from regions-of-interest (ROIs) showing general or specific adaptation effects. To further quantify adaptation strength, adaptation ratios [24] were calculated for the different adaptation conditions: estimated % signal change adaptation/no-adaptation. A ratio of one indicates no adaptation, whereas ratios between zero and one indicate different adaptation strengths and thus different levels of sensitivity to the varied property.

## Results

**Figure 4:**
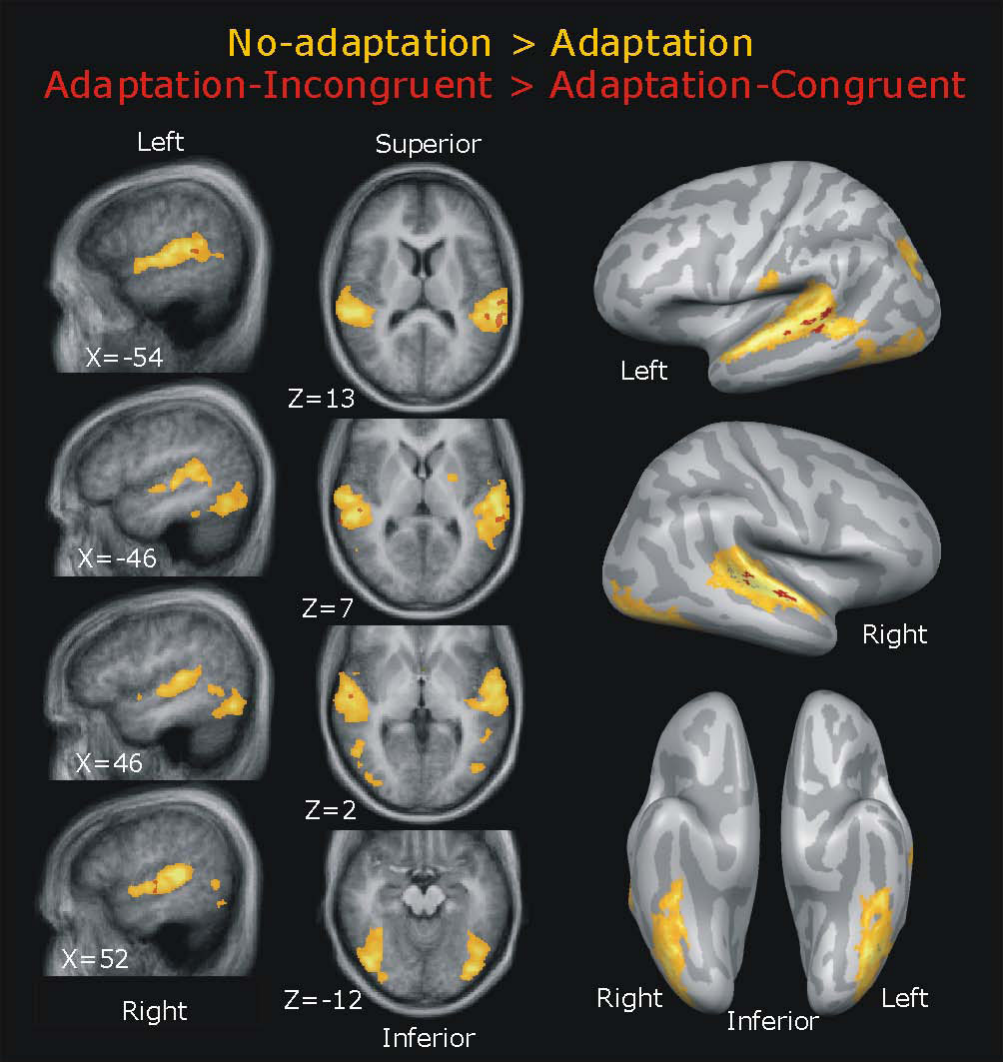
**Overview of general and specific adaptation effects**. Left panel: random-effects group maps (n = 16) of the No-adaptation vs. Adaptation (yellow) and Adaptation-Incongruent vs. Adaptation-Congruent (red) contrasts (cluster-level corrected at p < 0.05), projected on transversal and sagittal slices of the averaged anatomical image. Right panel: cortex-based aligned random-effects group maps (at the same threshold) on the inflated averaged cortical meshes of both hemispheres.

All subjects detected all auditory and visual target stimuli, which ensures a similar attention level during the adaptation and no-adaptation epochs. Figure 4 shows the statistical random-effects group maps of the two contrasts of interest: No-adaptation vs. Adaptation in yellow (*general adaptation*), and Adaptation-Incongruent vs. Adaptation-Congruent in red (*specific adaptation*). In the left panel, volume maps are shown on transversal and sagittal slices of the averaged anatomical image. Both contrasts were cluster-level corrected at p < 0.05 (No-adaptation vs. Adaptation: t_15 _= 3.75, initial voxel-level p-value < 0.0001, resulting minimum clustersize 123 mm^3^; Adaptation-Incongruent vs. Adaptation-Congruent: t_15 _= 3.2, initial voxel-level p-value < 0.006, resulting minimum clustersize 87 mm^3^). In the right panel, cortex-based aligned group maps are shown at the same t-values on the inflated averaged cortical meshes of both hemispheres, to provide a better overview of both maps.

A general adaptation effect was found bilaterally on the transverse temporal plane, middle (left) and superior temporal gyrus and sulcus (MTG/STG/STS), and the lateral and inferior occipito-temporal cortex (yellow map). Within this network, several smaller STS/STG clusters adapted stronger to Ad-C than to Ad-I, as reflected by a *weaker *fMRI response for Ad-C vs. Ad-I (red map). This indicates sensitivity to the manipulated audiovisual relation in those STS/STG clusters. No clusters showed this specific adaptation effect in the reverse direction. Table 1 summarizes the cluster volumes and Talairach coordinates of regions identified by the general and specific adaptation contrasts (volume data).

**Table 1:** Cluster volumes and Talairach coordinates of regions identified by the general and specific adaptation contrasts (volume data).

*Contrast *Brain area	Volume (mm^3^)	Center of mass (Talairach coordinates)			Peak of activity (Talairach coordinates)		
***General adaptation***		***x***	***y***	***z***	***x***	***y***	***z***

Left inferior occipito-temporal	8966	-41	-63	-11	-39	-76	-11

Left superior temporal	15218	-55	-26	9	-60	-28	7

Left superior/dorsal occipital	272	-28	-74	23	-27	-73	22

Right inferior occipito-temporal	11210	39	-62	-12	45	-67	11

Right superior temporal	12959	55	-24	7	54	-34	10

*Specific adaptation*							

Left superior temporal	87	-61	-24	15	-60	-25	16
	
	150	-52	36	11	-51	-34	13
	
	329	-60	-39	12	-60	-14	13

Right superior temporal	165	54	-15	1	54	-16	4

**Figure 5:**
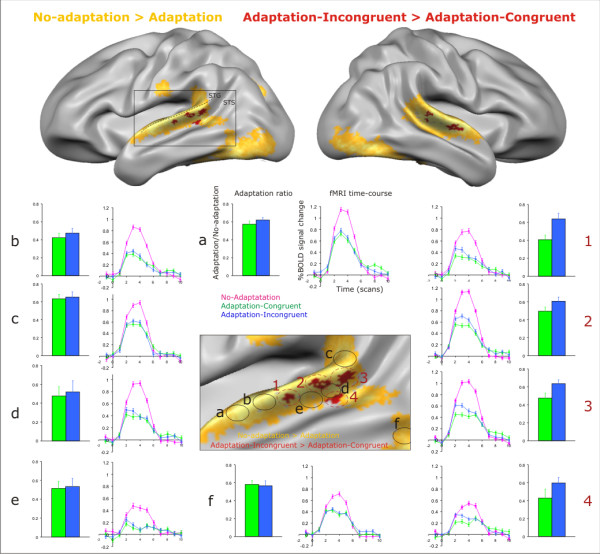
**Detailed localization and response profiles of superior temporal clusters adapting stronger to congruent than to incongruent audiovisual stimuli**. Cortex-based aligned group maps (n = 16) projected on the folded, shape-averaged cortical surface. Clusters showing stronger adaptation to congruent AV-stimuli (red) were localized on the upper bank of the STS and lower bank of the STG. Line graphs: averaged BOLD response time-courses extracted from encircled clusters. Bar graphs: adaptation ratio (estimated signal level adaptation/no-adaptation) in the same clusters. STS: superior temporal sulcus; STG: superior temporal gyrus.

For more precise macro-anatomical localization, figure 5 shows the same cortex-based aligned group maps on the folded, shape-averaged cortical surface. This reveals that clusters adapting stronger to Ad-C were located on the upper bank of the STS and the lower bank of the STG. The line graphs in figure 5 show the averaged BOLD response time-courses within ROIs extracted from both maps. The BOLD response to both adaptation conditions (blue and green lines) was equally reduced compared to the no-adaptation responses (pink lines) in the regions selected from the general adaptation map (ROIs *a *to *f*, graphs in centre/left). The time-courses from the significant regions of the specific adaptation contrast showed more suppression (i.e., adaptation) to Ad-C (green line) than to Ad-I (blue line), especially in the early phase of the response (ROIs *1 *to *4*, graphs on right). This immediate decrease for repeated stimuli is a typical finding for BOLD adaptation [31] and resembles simulated BOLD responses based on neuronal adaptation [24,32]. In addition, the bar graphs show the adaptation ratio in the same ROIs, calculated by dividing the estimated signal level during Ad-C and Ad-I by that during No-Ad. The adaptation ratios in the clusters showing specific adaptation (fig. 5, right bar graphs), also reveal stronger adaptation for congruent than for incongruent repeated letter-sound pairs, or in other words, partial recovery from adaptation during the incongruent pairs.

## Discussion

In the present study, we addressed the still open question of how content relatedness is encoded in the human superior temporal cortex (STS/STG). We used a variation of the fMR-adaptation design and relatively high-resolution voxels (2 × 2 × 2.5 mm^3^) to decrease susceptibility to potential BOLD spatial averaging and saturation confounds. We measured BOLD adaptation to repeated audiovisual (AV) stimuli (letter-speech sound pairs) and manipulated the associative relation between the visual (V) and auditory (A) inputs (congruent/incongruent pairs). Our key finding was that within a larger occipital-temporal network that adapted independently of the AV-relation (*general adaptation*), several smaller clusters distributed over STS/STG adapted stronger to repetitions of congruent than of incongruent AV-stimuli (*specific adaptation*). Since unisensory neurons are assumed to respond only to their preferred modality and therefore to be insensitive to the relation between the V and A inputs, this finding suggests that in these clusters, multisensory neurons are present that encode content relatedness by selectively responding to congruent AV-stimuli.

### General adaptation to letter-sound pairs

Voxels in which the fMRI response to either adaptation condition was significantly weaker than the unadapted response to letter-sound pairs were found on the transverse temporal plane, middle and superior temporal gyrus and sulcus (MTG/STG/STS), and the lateral/inferior occipital-temporal cortex (yellow maps in figures 3 and 4). Time-courses and adaptation ratios in figure 4 (left graphs) demonstrate that the BOLD response to both adaptation conditions was equally suppressed in this network. This indicates that neurons in these voxels respond and adapt to letters, speech sounds, *or *both. The revealed regions are consistent with other reports of letter and speech sound processing (e.g., [9,13,15,33,34]).

### Specific adaptation to the associative relation of letter-sound pairs

Within the network showing general adaptation, several clusters in STS/STG adapted stronger to repetitions of congruent than to repetitions of incongruent AV-pairs. Interestingly, no voxels showed the effect in the opposite direction. Assuming that stimuli initially eliciting the strongest response in a neuron also induce the largest response reduction by repetition [35], our findings support the prediction that subpopulations of multisensory neurons in distributed clusters in STS/STG encode audiovisual content-based relatedness by responding selectively to congruent stimulus pairs. This is in line with single-cell findings of congruency-selective audiovisual neurons in monkey STS [21].

Important for our interpretation is that we counterbalanced the unisensory inputs across both adaptation conditions: each letter and each speech sound exemplar was presented equally often in congruent and incongruent AV-pairs (see Materials and Methods, and fig. 2). This equalizes the averaged purely *unisensory *responses across both adaptation conditions, so the demonstrated differential adaptation strengths can be attributed to sensitivity to the varied AV-relation (and not to different adaptation strengths to different stimulus exemplars). Therefore, we propose that the observed adaptation differences indicate selectivity to congruent AV-stimuli on the level of multisensory neurons.

As adaptation is thought to reflect selectivity at the input rather than at the output level of neurons [32], several speculations towards a neuronal mechanisms for congruency-selectivity can be made: either *more *synaptic inputs converge on multisensory STS/STG neurons for congruent AV-inputs compared to incongruent inputs, or in *different *excitatory/inhibitory convergence patterns [36], or *only *congruent inputs converge. In any of these mechanisms, these multisensory neurons will adapt stronger (or exclusively) to congruent AV-stimuli.

### The neural mechanism for letter-speech sound integration

Interestingly, the present results reveal a response pattern that is different from our previous studies on letter-sound integration using the same stimuli but non-repeated presentation. In these studies, congruency effects were most pronounced in early stages of the auditory cortex, and less consistently observed in STS/STG [13,15]. The STS/STG did show a heteromodal response pattern and enhanced responses to AV stimuli compared to both A and V responses. We therefore interpreted the STS/STG as integrator, and the congruency effect in auditory cortex as feedback modulation, which was supported by effective connectivity analyses [37]. However, it remained unresolved why the STS/STG in that case did not show sensitivity to the congruency of the letter-sound pairs, as it is assumed to provide differential feedback. The present results complement these previous findings by showing that distributed clusters in STS/STG clearly are sensitive to content congruency, expressed by differential adaptation strengths rather than BOLD amplitude per se. The latter might not be sensitive enough to reflect these differences due to saturation effects, as outlined in the introduction. But why was adaptation in early auditory regions not sensitive to congruency in the present study? The strong congruency effect in auditory cortex observed during non-repeated stimulation may be the result of amplification of neural activity (in both directions: enhancement and suppression) by the feedback from STS [38], which is likely to be cancelled out when STS activity is suppressed by stimulus repetitions.

### Organization of human multisensory superior temporal cortex

The present results suggest that several clusters within the human STS/STG contain multisensory neuronal subpopulations that are sensitive to the associative relation between audiovisual inputs. Using cortex-based alignment of anatomical and functional data, we were able to localize these clusters precisely and reliably on the upper bank of the STS and lower bank of the STG (approximate cluster area: 4, 6, 13, 5 mm^2 ^(left); 3, 5, 23 mm^2 ^(right)), which is consistent with the location of multisensory neurons in the superior temporal polysensory area (STP) in monkeys (e.g., [19,20]; see [5] for review). Since the fMRI signal in the incongruent adaptation condition recovered only partially, these clusters are likely to be composed of a mixture of uni- and multisensory neuronal subpopulations, rather than only of multisensory neurons. Our results therefore corroborate the reported patchy distribution of unisensory and multisensory neuronal subpopulations in human STS [22] which was recently supported by electrophysiology in macaques [23], and the neuronal organization within transitional multisensory zones in rats [39]. Moreover, even though the patchy organization of uni- and multisensory neurons may differ between individuals [22], there seems to be enough overlap of voxels containing multisensory clusters to be robustly revealed on the group-level using macro-anatomical intersubject alignment methods.

### fMR-A as a new approach to study human multisensory integration?

The present study shows the feasibility of fMR-adaptation to provide insights in human multisensory integration by circumventing some of the limitations imposed by the coarse spatial resolution and limited dynamic range of the fMRI signal. This is much needed since other approaches to deal with results from large neuronal samples, such as the super-additivity metric, are not satisfactory [28,40]. Using the current design, other stimulus types and other manipulations of the multisensory relation (onset, location) can be investigated in future studies. Other fMR-A designs can be employed as well, for example, it would be very interesting to present visual and auditory stimuli in alternation instead of simultaneous, which has been used to investigate feature integration *within *modalities [41] and is the more typical fMR-A design (see introduction). In such a design, cross-adaptation between modalities might reveal multisensory convergence on the neuronal level in more detail. However, there are several potential pitfalls for such designs (see also [28]), which all result from the putatively mixed unisensory-multisensory organization of "multisensory" brain regions like STS/STG [22]. One complication is the observation that neurons adapt despite intervening stimuli [31], so stimulus repetitions in alternating modalities will also adapt unisensory neurons, although probably to a weaker extent. Another problem is that a cross-modal repetition (e.g., visual-auditory) may suppress activity of multisensory neurons, but will also activate new pools of unisensory neurons (in this example: auditory) in the same voxel, which may counteract the cross-modal suppression.

It should also be kept in mind that the exact neuronal mechanism underlying BOLD adaptation is still uncertain [31,32,42,43]. For example, a factor that complicates the interpretation of BOLD adaptation results is that it may reflect only the outcome of more complex changes within networks, such as inherited adaptation from distant regions disturbing the normally balanced input [43]. Our data show the specific adaptation effect exclusively in STS/STG clusters; therefore it seems unlikely that this pattern is inherited from upstream sensory regions.

## Conclusions

We demonstrated that BOLD adaptation in distributed superior temporal clusters is sensitive to the associative relation between visual and auditory inputs, which indicates the presence of multisensory neuronal subpopulations in human STS/STG that encode content congruency. These findings extend our previously revealed mechanism for the integration of letters and speech sounds and demonstrate that fMR-A is sensitive to multisensory congruency effects that may not be revealed in BOLD amplitude per se.

## Authors' contributions

NMvA conceived of the study, developed the design, conducted the measurements, performed the statistical analysis and drafted the manuscript. VCB contributed to developing the design, participated in conducting the measurements and helped to draft the manuscript. LB contributed to conception of the study, participated in coordination and helped to draft the manuscript. RG contributed to conception and design, advising on statistical analysis and contributing the appropriate analysis tools. All authors read and approved the final manuscript.
